# Developing a Gudermannian neural network for solving the Painlevé model-II in the context of nonlinear optics

**DOI:** 10.1038/s41598-026-43643-0

**Published:** 2026-04-05

**Authors:** Sundas Faisal, Zulqurnain Sabir, Samra Urooj Khan, Muhammad Aamir, Krzysztof A. Cyran

**Affiliations:** 1 Department of Graphics, Computer Vision and Digital Systems, Silesian University of Technology, Gliwice, Poland; 2 Department of Computer Science and Mathematics, Lebanese American University, Beirut, Lebanon; 3 Faculty of Electrical and Electronic Engineering, Universiti Malaysia Pahang Al-Sultan Abdullah, Pekan, Pahang, 26600 Malaysia; 4 Department of Computer Science, University of Oxford, Wolfson building, Parks Road, Oxford, OX1 3QG UK

**Keywords:** Painlevé model-II, Nonlinear optics, Gudermannian function, Neural networks, Particle swarm optimization, Sequential quadratic programming, Engineering, Mathematics and computing

## Abstract

A design of Gudermannian neural network (GNN) is executed for the numerical results of the Painlevé model-II in the context of nonlinear optics (PM-II-NO). One of the forms of artificial neural networks is GNN, which uses the Gudermannian function (GF) as a merit function. This function has a nonlinearity that performs a complicated association in inputs and outputs. The design of the merit function is performed based on the differential PM-II-NO and boundary conditions, which is optimized further using the hybrid of the global search particle swarm optimization (PSO) and local search sequential quadratic programming (SQP), i.e., PSO-SQP. The algorithm’s accuracy is perceived via matching of obtained and reference database solutions, and insignificant performance of absolute error. Furthermore, the statistical investigations based on multiple independent executions are performed in order to check the reliability of the scheme by various tests, e.g., mean square error, semi inter-quartile range, and Theil inequality coefficient in order to present the significance and reliability of the designed GNN-PSO-SQP for the PM-II-NO. The comparison of the proposed results taking 5, 15, and 45 numbers of neurons and literature results is also presented to perform the neuron analysis of this study. This designed neural network is mainly valuable to solve differential systems, such as PM-II-NO, which can competently estimate the solutions by applying the GF properties.

## Introduction

At the beginning of the twentieth century, Painlevé developed all kinds of Painlevé models (PMs). Subsequently, another of his pupils, Gambier, developed a list of these formulas. These systems follow the Painlevé material goods and feature with no adaptable singularities of outcomes via present posts ^1^. PMs indicate a class of nonlinear ordinary models, which is extensively implemented in mathematics, engineering, and physics. It is also widely applied in the arbitrary matrix theory, Frobenius manifolds, dynamical systems, orthogonal polynomials, reduced partial integro models, enumerative algebraic geometry, and number theory ^2^. The six PMs (P-1 to P-6), often known as the Painlevé transcendent (PT) in nature, are crucial items that arise from studies of compatible systems that display various characteristics, such as soliton solutions, Lax pairs, and algebraic-geometric structures ^3^. Although the PT cannot be reduced to ordinary distinct capacities, research does give traditional solutions of PMs utilizing specialized variables, such as Hermite, airy, hypergeometric, and Bessel functions ^4^. In the area of mathematics, PMs are connected to algebraic as well as differential geometry, whereas they indicate a dynamic role to exist the perilous sensations in physics, mainly in the theory of quantum field and phase evolutions ^5^. The actual-life phenomena, such as population/fluid mechanics and optical science, have been modeled using these formulae. In the study of nonlinear optics, PMs designate the comportment of optical pulses and rays in frequent media ^6^. In general, P-2 and P-3 models deliver a banquet of ultra-short beats based on optical characters. These models are widely implemented to detect the effects of cross-phase inflection, self-phase dispersion, and inflection that are used to examine the complex sensations, e.g., pulse density, optical shock waves, and soliton formation ^7^. Furthermore, P-6 has studied the beam spread and optical twister solitons based on the nonlinear media together with the variable refractive index.

Mathematical systems represent a vital role in describing and understanding the intricate phenomena in different areas, like biology, engineering, economics, and physics. The mathematical models have been applied based on mathematical techniques and languages in order to signify the practical systems, which make it possible to predict, examine, and optimize their presentation. The significance of mathematical systems represents their aptitude to simplify intricate systems, facilitate imitations, and perform predictions about future consequences. Based on mathematical systems, engineers and scientists can perform hypotheses, and take decisions deprived of trusting on impractical or expensive experiments. The mathematical form of the systems has been performed in abundant innovations, like weather prediction and growth of population to optimize intricate models and recognize the performance of subatomic elements. There are several types of mathematical systems, e.g., empirical, deterministic, and stochastic, with each of its own merits and demerits. Empirical systems depend on data-driven schemes to recognize associations in variables. Deterministic systems rely on differential equations, which give accurate predictions. On the other hand, stochastic systems handle randomness and uncertainty. The mathematical models have been used in various submissions, e.g., drying processes of vegetables and fruits ^8^, evolving nanoparticle of cancer system based drug delivery ^9^, the fractional mathematical system of Tumour metastasis and invasion ^10^, a mathematical multi-objective model based on the water management together with environmental effects ^11^, dynamics of coronavirus system with isolation, quarantine, and ecological viral load ^12^, chloride-induced reinforcement corrosion rate ^13^, echelon supply chain system ^14^, and malaria disease with insecticides and treatment ^15^.

The purpose of this study is to onstruct a novel Gudermannian neural network (GNN) together with the optimization of global search particle swarm optimization (PSO) and local search sequential quadratic programming (SQP), i.e., GNN-PSO-SQP, to get the numerical performances of Painlevé model-II in the context of nonlinear optics (PM-II-NO). The mathematical form of the PM-II-NO along with its initial conditions (ICs) is given as ^16^:

1 $$\left\{\begin{array}{c}\frac{{d}^{2}p\left(u\right)}{d{u}^{2}}=2{p}^{3}\left(u\right)+up\left(u\right)+\lambda,\\p\left(0\right)=\frac{dp\left(0\right)}{du}=0.\end{array}\right.$$

The model presented in the above equation mainly appears in nonlinear optics, which transforms the intricate amplitude using electric field propagation in an optical fiber. These results are specified via P-II based on appropriate transformations. The system using P-II has been broadly applied by various researchers in different approaches ^17,18^. In the literature, it is clear that PM-II-NO has not been exploited numerically before by using the GNN-PSO-SQP. In recent decades, the stochastic computing schemes have been used in frequent submissions, e.g., lungs cancer operation model ^19^, cholera disease model ^20^, monkeypox transmission system ^21^, Zika model with reservoirs and human movement ^22^, Buruli ulcer and cholera model ^23^, divorced dynamics in social networks ^24^, cattle skin disease model ^25^, epidemiological model of the mumps virus ^26^, and Maxwell fluid model ^27^. Moreover, the solutions of the PM-II-NO has currently been presented by using the design of heuristic Morlet wavelet neural network ^28^. A few of the novel insights of this work are given as:

- A mathematical form of the PM-II-NO is numerically handled effectively by applying one of the competent GNN-PSO-SQP processes.
- The investigations based on the GNN-PSO-SQP are explored first time for solving PM-II-NO.
- The test of optimization is performed using the combination of global search PSO and local search SQP.
- A merit function is produced based on the differential PM-II-NO and boundary conditions.
- The comparison of the proposed and literature solutions is also provided to authenticate the exactness of the designed solver.
- The correctness of the scheme based on GNN-PSO-SQP is performed by comparing the obtained and reference outcomes.
- Some statistical tests are also programmatic to authenticate the precision of designed GNN-PSO-SQP scheme.

The remaining paper sections are given as follows: Section 2 reports the design of the GNN-PSO-SQP procedure, the discussions of the results of differential PM-II-NO are provided in Section 3, and the conclusions are listed in Section 4.

## Designed methodology: GNN-PSO-SQP

The current section designates a novel GNN-PSO-SQP procedure for the differential PM-II-NO in two stages as:

- Construction of a merit function is performed using the differential PM-II-NO.
- Optimization is performed by using PSO-SQP.

### Structure of GNN

A merit Gudermannian function (GF) having the process of optimization based on PSO-SQP is presented. GF is one of the mathematical functions which applies the merit function in the process of neural networks. It has exclusive properties that permit it to present nonlinearity, which allows the system to present and learn complicated associations in inputs and outputs. GF in the process of a neural network provides the support to the system to seizure indirect designs and subtleties in data, mainly in different submissions connecting differential systems or distinct functions. There are several research studies that have been exploited based on the application of GF when used as a neural network to present the solutions of a precise model, like the Lane-Emden system. The neural network structure is presented as:

2 $$\widehat{p}\left(u\right)={\sum}_{i=1}^{m}{k}_{i}Q({w}_{i}u+{d}_{i}),$$

$$\frac{{d}^{(n)}}{d{u}^{(n)}}\widehat{p}\left(u\right)={\sum}_{i=1}^{m}{k}_{i}\frac{{d}^{(n)}}{d{u}^{(n)}}Q({w}_{i}u+{d}_{i}).$$

In Eq. (2), *m* represents the neurons, and the unknown weights are $\boldsymbol{W}=[k,w,i]$, i.e., $k=\left[{k}_{1},{k}_{2},...,{k}_{m}\right],w=\left[{w}_{1},{w}_{2},...,{w}_{m}\right]\mathrm{and}\quad d=[{d}_{1},{d}_{2},...,{d}_{m}].$ To solve the differential PM-II-NO, GF has never been designed before; the mathematical form of GF is shown as:

3 $$Q\left(u\right)=2{{tan}}^{-1}\left[{exp}(u)\right]-\frac{1}{2}\pi.$$

Equation (2) is updated using the above expression (3) as:

$$\widehat{p}\left(u\right)={\sum}_{i=1}^{m}{k}_{i}(2{{tan}}^{-1}{e}^{({w}_{i}u+{d}_{i})}-\frac{1}{2}\pi),$$

4 $$\frac{d}{dz}\widehat{p}\left(u\right)={\sum}_{i=1}^{m}2{n}_{i}{w}_{i}\left(\frac{{e}^{({w}_{i}u+{d}_{i})}}{1+{\left({e}^{({w}_{i}u+{d}_{i})}\right)}^{2}}\right),$$

$$\frac{{d}^{2}}{d{z}^{2}}\widehat{p}\left(u\right)={\sum}_{i=1}^{m}2{k}_{i}{w}_{i}^{2}\left(\frac{{e}^{({w}_{i}u+{d}_{i})}}{1+{\left({e}^{({w}_{i}u+{d}_{i})}\right)}^{2}}-\frac{2{e}^{({w}_{i}u+{d}_{i}{)}^{3}}}{{\left(1+{\left({e}^{({w}_{i}u+{d}_{i})}\right)}^{2}\right)}^{2}}\right).$$

A merit function ${E}_{Fit}$ is designed as:

5 $${E}_{Fit}={E}_{Fit-1}+{E}_{Fit-2}.$$

where ${E}_{Fit-1}$ and ${E}_{Fit-2}$ signify the error function based on differential PM-II-NO and ICs as:

6 $${E}_{Fit-1}=\frac{1}{N}{\sum}_{i=1}^{m}\left(\frac{{d}^{2}}{{dx}^{2}}{\widehat{p}}_{i}-2{\widehat{p}}_{i}^{3}-{u}_{i}{\widehat{p}}_{i}-\lambda\right),0\le{u}_{i}\le1.$$

and

7 $${E}_{Fit-2}=\frac{1}{2}{\left({\widehat{p}}_{0}\right)}^{2}+\frac{1}{2}{\left(\frac{d}{du}{\widehat{p}}_{N}\right)}^{2}.$$

### Optimization: PSO-SQP

The optimization performances based on PSO-SQP are accessible for the differential PM-II-NO. The description of PSO and SQP is given as:

PSO is one of the population forms of stochastic optimization scheme, which is inspired by the social comportment of fish schooling or birds flocking. The scheme contains a swarm-based of particles, which signify a possible outcome to the optimization model. All particles have a velocity, position, and values of the fitness that are assessed by applying the cost function. The particles exchange via search space, which is influenced through their individual experience and its neighbors to perform optimal results. PSO informs the position and velocity of every particle using its particular optimal (poptimal) position and the global optimal (goptimal) position established through the swarm. The upgradation of velocity is performed through the present velocity of particles, and its distance via goptimal and poptimal. The upgrade of position is then performed using the new form of the velocity. This scheme is iteratively repetitive by permitting the swarm to converge to the best point. PSO is recognized due to its flexibility, simplicity, and aptitude to deal with complicated optimization systems. It has been extensively implemented in numerous areas, like finance, engineering, and computer science, which is used to present the optimization models, e.g., parameter approximation, function optimization, and feature selection. Its benefits comprise quick convergence, fast execution, and aptitude to deal with non-convex and nonlinear problems. Moreover, PSO can undergo stagnation and premature convergence, while its presentation can be profound in constraint settings. A number of PSO variants have been planned to deal with these confines and increase its routine. Currently, PSO has been used in many applications, e.g., electrical power system ^29^, large scale numerical optimization and its engineering applications ^30^, fuzzy controlled servo systems ^31^, multiple attribute decision making in vertical handover in heterogenous wireless networks ^32^, and vehicle routing problem with time windows ^33^.

The best global values of global PSO are used as an input in the local search SQP for solving the differential PM-II-NO. SQP is one of the numerically powerful schemes implemented to present the solutions of the constrained problems based on optimization. SQP iteratively presents a progression of quadratic programming subproblems, which is used to approximate the original nonlinear model. At each generation, SQP performs the method of linearization on the nonlinear limitations and builds a quadratic system of the cost function that is then optimized to perform a novel iteration. This procedure continues till convergence to an outcome is obtained. SQP is broadly implemented in numerous areas, e.g., design of engineering, machine learning, and finance, because of its capability to tackle intricate nonlinear models with constraints-based equality and inequality. SQP is recognized due to its quick rate of convergence and sturdiness, which makes it a general high-quality method to solve the models based large-scale optimization models. Moreover, this process is computationally exclusive, mainly for those models which have various constraints or variables and involves cautious algorithmic parameters tuning to guarantee competent performance. Currently, SQP has been reported in various applications, e.g., constrained multiobjective optimization ^34^, large-scale constrained optimization ^35^, facies classification ^36^, nonlinear complementarity constraints ^37^, and structural design problems ^38^.

The hybridization of global search (PSO) and local search (SQP) is performed for presenting the solutions of the differential PM-II-NO, because global search obtains a good primary point, whereas local exploitation upgrades it to meet to a more precise optimum. This synergy progresses routinely by joining global search’s aptitude to evade local optima with local search’s rapid convergence, exploiting their assets to discover an improved result.

### GNN limitations

**Fig. 1:**
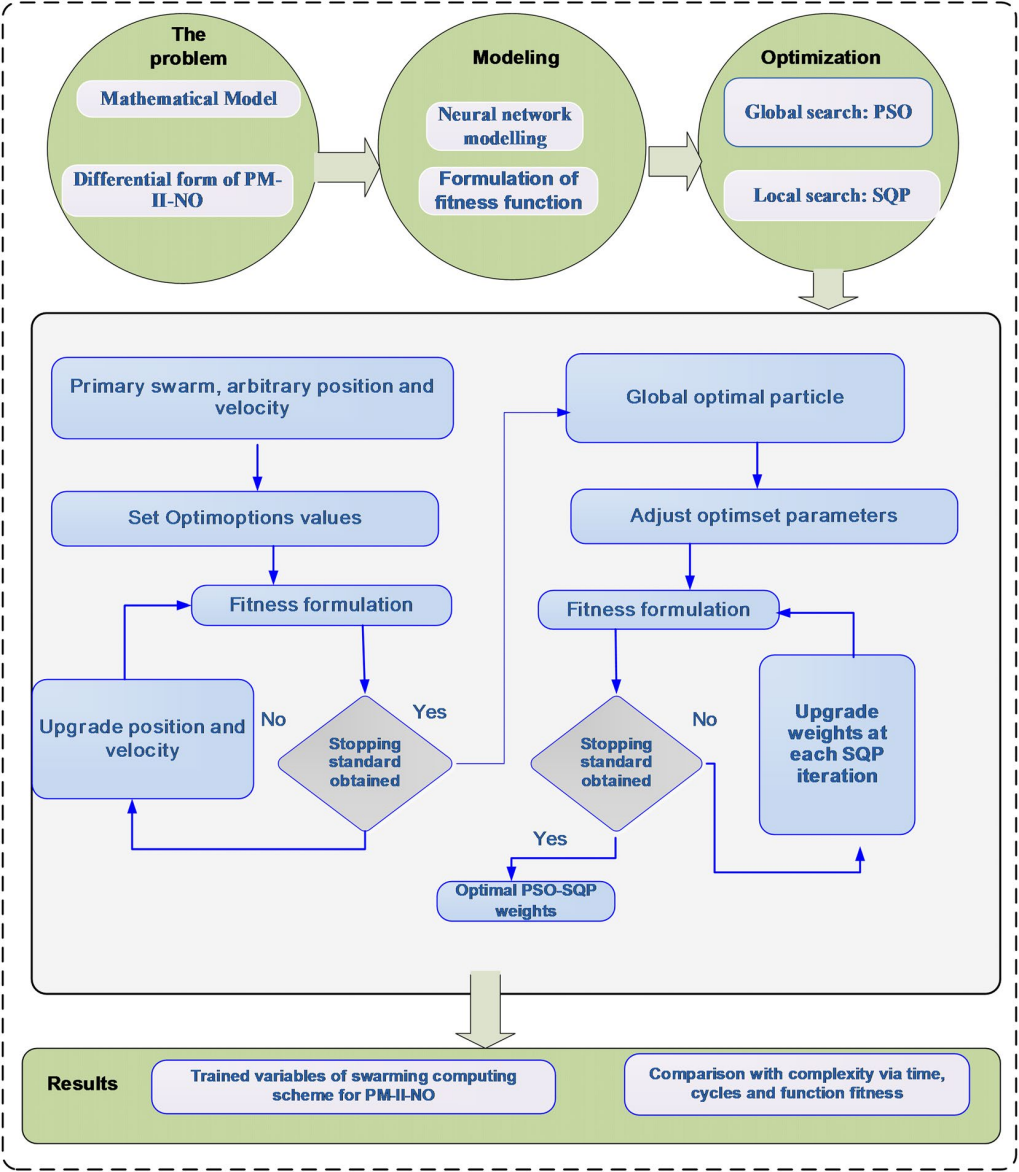
Proposed structure based on GNN-PSO-SQP structure for differential PM-II-NO.

GNN, while auspicious to present the solutions of various complicated models, has a number of limitations. A major limitation is the prerequisite for cautious tuning of the parameter, as the system performance mainly depends on the suitable selection of the parameters. In addition, the rate of constancy and convergence of GNN is performed as sensitive to ICs and the model complexity being resolved. These influences can distress the capability of the system to detect precisely the fundamental model’s dynamics. One more limitation of GNN is the prospective for underfitting or overfitting, mainly if the structure of the system is not well organized with the explicit model. The cost of complexity in order to train the GNN may also become a limiting issue, generally for those problems which has a large-scale or by applying intricate optimization schemes. Moreover, the outcomes’ interpretability can be stimulating because of its nonlinear nature-based GF, which makes it problematic to appreciate the relations in inputs and outputs. The construction of the GNN-PSO-SQP for solving the differential PM-II-NO is illustrated in Fig. 1.

## Statistical procedures

The procedures based on the mean square error (MSE), semi inter-quartile range (SIQR), and Theil inequality coefficient (TIC) are presented in this section. The process of MSE is one of the metrics to formulate the presentation of predictive systems, mainly in regression procedures. It computes the regularity of the squared differences in projected and authentic performances, that shows a portion of the precision of the system. The small values of MSE present improved fitness of the model, whereas a greater MSE proposes superior deviations in estimates and actual performances. MSE is one of the popular selections because of its interpretability and minimalism. It is widely applied as a cost function to train machine learning systems, where the motive is to reduce the MSE to accomplish the best value. Moreover, MSE has a number of boundaries, e.g., understanding outliers along with its postulation of identical positions for each error. Although these boundaries, MSE vestiges an important metric to assess and compare the presentation of different systems, which provide respected understandings into their prognostic competences.

SIQR is a ration of statistical distribution, which performs as 1/2 the difference in the 3rd quartile (*q*_3_) and 1st quartile (*q*_1_) of the data. It signifies the data spreading to the middle 50%, which provides a vision into the data’s inconsistency, whereas it performs less sensitivity to outliers in comparison with other procedures based on standard deviation (STD). SIQR is specifically applied for skewed flows or datasets with extreme performances, where it suggests a more accurate spread portion. SIQR is appreciated in data examination and expressive statistics, which denote a clear sign of the central propensity and inconsistency of data. It is widely applied in combination with the average to signify inclusive consideration of the features of data.

The TIC is recognized to perform predictive systems, which are mainly applied in forecasting. It evaluates the prediction accurateness through associating them with an ingenious forecast, like as an arbitrary no-change or walk system. TIC statistics compute the relation of the prediction error-based root mean square of the system based on the innocent prediction. The performance of TIC under 1 designates that the predictions of the system, which is improved than naive prediction, whereas a performance higher than 1 proposes that the prediction of the system is inferior. The TIC is applicable to assess the value of various predicting systems in order to classify the precise prediction of the system. It supports controlling whether a prediction of the system is suggestively better over a simple scheme. TIC is widely applied in several submissions, including finance, econometrics, and other areas where prediction is considered complex. The mathematical forms of MSE, SIQR, and TIC are given as:

8 $$MSE={\sum}_{j=1}^{k}{\left({p}_{j}-{\widehat{p}}_{j}\right)}^{2}.$$

9 $$\mathrm{SIQR=}-0.5\left({q}_{1}-{q}_{3}\right).$$

10 $$\mathrm{TIC=}\frac{\sqrt{\frac{1}{n}{\sum}_{j=1}^{k}{\left({p}_{j}-{\widehat{p}}_{j}\right)}^{2}}}{\left(\sqrt{\frac{1}{n}{\sum}_{j=1}^{k}{p}_{j}^{2}}+\sqrt{\frac{1}{n}{\sum}_{j=1}^{k}{\widehat{p}}_{j}^{2}}\right)}.$$

In the above system, ${p}_{j}$ and ${\widehat{p}}_{j}$ are the reference and proposed results.

## Comparison of the results

This section shows the comparison of the results based on the PM-II-NO by taking heuristic Morlet wavelet neural network ^28^ and designing GNN-PSO-SQP structure. The comparison of the Adam method, reference Morlet wavelet neural network, and the designed GNN-PSO-SQP structure by taking 5, 15 and 45 numbers of neurons is presented. It is observed that the Adam and reference solutions matched with the designed procedure by taking 5, 15, and 45 neurons, which shows that 15 and 45 neurons clearly matched with the Adam and reference solutions in comparison with the 5 neurons. It is observed that by enhancing the number of neurons, the accuracy of the solver is enhanced. Moreover, this comparison gives confidence to the authors to design the GNN-PSO-SQP structure for solving the PM-II-NO. The comparison of the results is presented in Table 1.

**Table 1:** Comparison of the proposed, and literature results.

u	Adam method	Reference [28]	Proposed results (5 neurons)	Proposed results (15 neurons)	Proposed results (45 neurons)
0	0.00000000000	0.00001577245	0.08068473079	0.00000025653	0.00000000000
0.1	−0.01000051392	−0.00998248561	0.08193564822	−0.01000087055	−0.01000069416
0.2	−0.04001611694	−0.03999917230	0.06423374363	−0.04001738992	−0.04001680238
0.3	−0.09012393836	−0.09011084677	0.02656320815	−0.09012552300	−0.09012482956
0.4	−0.16053616667	−0.16052315203	−0.03059532678	−0.16053879076	−0.16053721177
0.5	−0.25170712673	−0.25168777583	−0.10674320038	−0.25171111969	−0.25170865066
0.6	−0.36451508316	−0.36449013538	−0.20236405942	−0.36451935176	−0.36451703604
0.7	−0.50058368709	−0.50056241658	−0.31906442047	−0.50058834482	−0.50058577225
0.8	−0.66285015917	−0.66283456789	−0.45948548393	−0.66285676174	−0.66285279278
0.9	−0.85657424342	−0.85654965641	−0.62723448893	−0.85658141022	−0.85657713633
1	−1.09118520112	−1.09116426928	−0.82688230714	−1.09119552730	−1.09119032196

## Simulations of the outputs

The current section presents the simulation results of the differential PM-II-NO based on three cases by applying the structure of GNN-PSO-SQP.

### Example 1

Suppose $\lambda=-2$, while the neurons are selected as 5:

11 $$\left\{\begin{array}{c}\frac{{d}^{2}p\left(u\right)}{d{u}^{2}}=2{p}^{3}\left(u\right)+up\left(u\right)-2,\\p\left(0\right)=\frac{dp\left(0\right)}{du}=0.\end{array}\right.$$

A fitness function is presented as:

12 $${E}_{Fit}=\frac{1}{5}{\left(\frac{{d}^{2}}{d{u}^{2}}{\widehat{p}}_{i}-2{\widehat{p}}_{i}^{3}-{u}_{i}{\widehat{p}}_{i}+2)\right)}^{2}+\frac{1}{2}\left(({\widehat{p}}_{0}{)}^{2}+{\left(\frac{d}{du}{\widehat{p}}_{0}\right)}^{2}\right).$$

The numerical performances using the optimization of GNN-PSO-SQP are presented for the optimization of Eq. (12). These weights characterise the fit parameters, which perform the suitable outcomes approximation. Five runs in Example 1 are accomplished that presents the calculations for numerous neuronal calculations to confirm the recommended scheme for defining the neurons based on optimal fitting. The GF is applied as an error function that is accomplished to prompt various series results as:

13 $$\widehat{p}\left(u\right)={\sum}_{i=1}^{5}{k}_{i}\left(2{{tan}}^{-1}{e}^{\left({w}_{i}u+{j}_{i}\right)}-\frac{1}{2}\pi\right).$$

The efficient form of above system is given as:

$$\begin{aligned}\widehat{p}\left(u\right)=0.0011\left(2{{tan}}^{-1}{e}^{\left(-6.4283u+5.6396\right)}-\frac{1}{2}\pi\right)\\-1.9342\left(2{{tan}}^{-1}{e}^{\left(1.1634u-1.6160\right)}-\frac{1}{2}\pi\right)\\+3.1144\left(2{{tan}}^{-1}{e}^{\left(-14.8891u-20.00\right)}-\frac{1}{2}\pi\right)\\+0.7991\left(2{{tan}}^{-1}{e}^{\left(-5.7104u+8.1615\right)}-\frac{1}{2}\pi\right)\end{aligned}$$

14 $$-1.2718\left(2{{tan}}^{-1}{e}^{\left(-1.4033u-1.3556\right)}-\frac{1}{2}\pi\right).$$

**Fig. 2:**
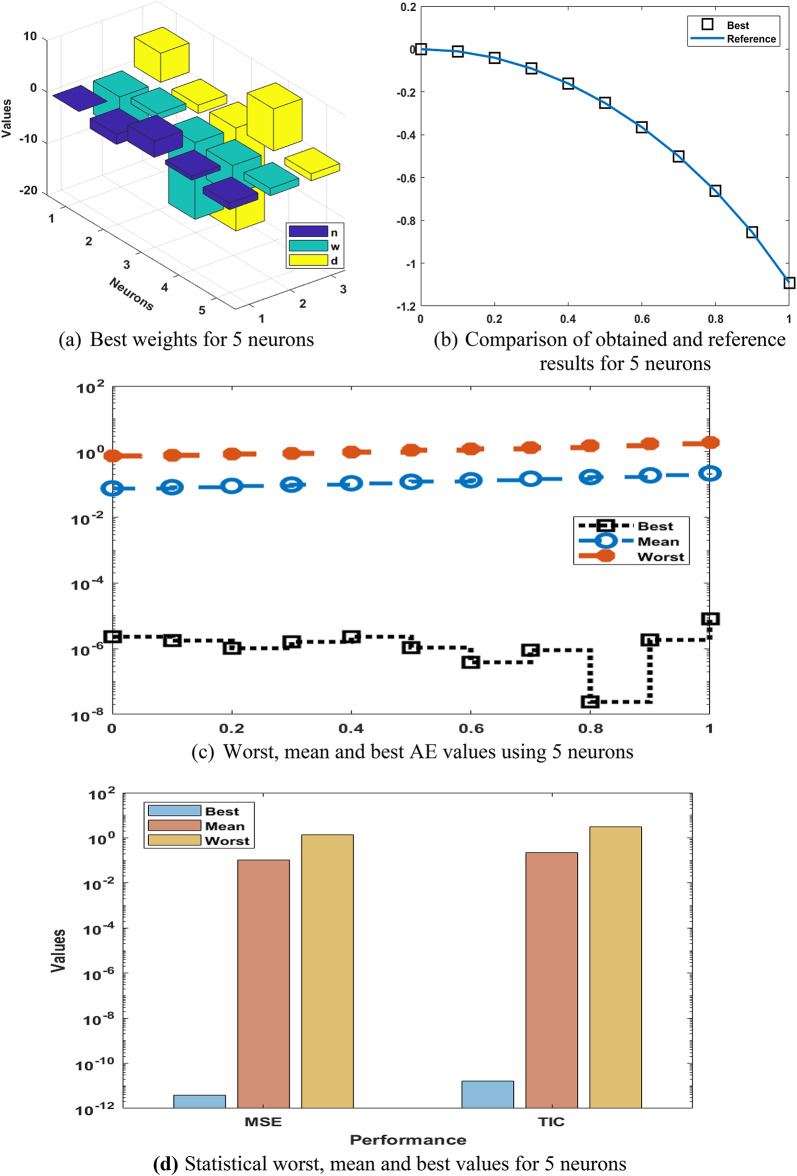
Best weights, output comparisons, AE, and numerical outcomes to solve the differential PM-II-NO using GNN-PSO-SQP based 5 neurons.

The values of the best weights, result comparisons, performances of absolute error (AE), and statistical outcomes are available in Fig. 2. The optimal weights are provided in Fig. 2(a) using 5 executions. The matching of reference and best outcomes is performed in Fig. 2(b). These results in five executions are matched with reference outputs, which authenticates the competence of the designed scheme to present the numerical results of differential PM-II-NO. Figure 2(c) presents the AE performances using the worst, mean, and best solutions. It is observed that most of the AE are performed around 10^− 06^, while some of the best values lie as 10^− 07^, whereas the mean and worst AE lie as 10^− 01^ to 10^− 02^. The mathematical form of the AE is calculated by using this formula: $\mathrm{AE=}\left|\left({p}_{i}-{\widehat{p}}_{i}\right)\right|$.

**Fig. 3:**
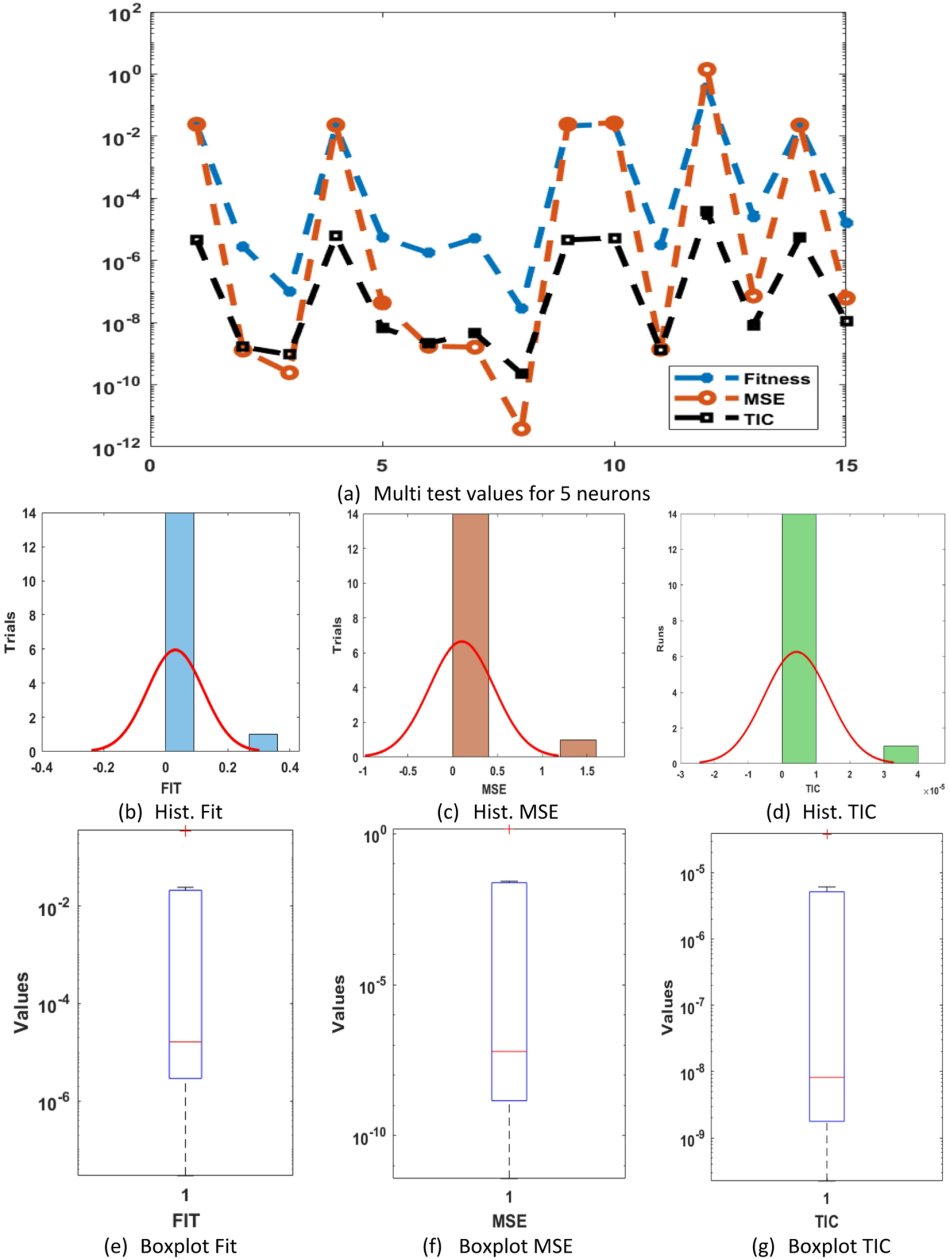
Multi test values, Hist. and boxplot for the differential PM-II-NO using GNN-PSO-SQP based 5 neurons.

These redcible AE signify the exactness of GNN-PSO-SQP for solving the differential PM-II-NO. The statistical performance-based worst, best, and mean using 5 neurons are accessible in Fig. 2(d). The statistical values are shown for three operators: MSE, SIQR, and TIC. The worst, mean, and best MSE values are noticed nearer to 10^− 01^, 10^− 02^, and 10^–12^. The worst, mean, and best TIC performances are dignified as 10^− 00^, 10^− 01^, and 10^–11^. These statistical procedures designate the sustenance to evaluate the performance of neural network. Based on the comparison of proposed and reference solutions, these statistical assessments signify the vision into the model’s precision, correctness, and steadiness. Fig. 3 presents the statistical values, boxplot, and histogram (Hist.) for the differential PM-II-NO. It is designated that a number of performances using MSE, SIQR, and TIC are shown into sensible percentages that indicate the consistency of the proposed GNN-PSO-SQP for presenting the solutions of differential PM-II-NO.

Table 2 signifies the performances of statistical operators using various operators’ minimum (Min), MD, mean, SIR, and STD. These small and negligible performances update the dependability of the designed GNN-PSO-SQP based 5 neurons.

**Table 2:** Multi statistical values using GNN-PSO-SQP for differential PM-II-NO.

Mode	$\widehat{p}\left(u\right)$										
	0	0.1	0.2	0.3	0.4	0.5	0.6	0.7	0.8	0.9	1
Min	2 × 10^− 6^	8 × 10^− 7^	1 × 10^− 6^	2 × 10^− 6^	2 × 10^− 6^	1 × 10^− 6^	4 × 10^− 7^	9 × 10^− 7^	2 × 10^− 8^	2 × 10^− 6^	7 × 10^− 6^
MD	7 × 10^− 5^	6 × 10^− 5^	8 × 10^− 5^	1 × 10^− 4^	2 × 10^− 4^	3 × 10^− 4^	2 × 10^− 4^	3 × 10^− 4^	3 × 10^− 4^	4 × 10^− 4^	4 × 10^− 4^
Mean	8 × 10^− 2^	8 × 10^− 2^	9 × 10^− 2^	1 × 10^− 1^	1 × 10^− 1^	1 × 10^− 1^	1 × 10^− 1^	2 × 10^− 1^	2 × 10^− 1^	2 × 10^− 1^	2 × 10^− 1^
SIR	4 × 10^− 2^	5 × 10^− 2^	5 × 10^− 2^	6 × 10^− 2^	6 × 10^− 2^	7 × 10^− 2^	8 × 10^− 2^	9 × 10^− 2^	1 × 10^− 1^	1 × 10^− 1^	1 × 10^− 1^
STD	2 × 10^− 1^	2 × 10^− 1^	2 × 10^− 1^	2 × 10^− 1^	3 × 10^− 1^	3 × 10^− 1^	3 × 10^− 1^	3 × 10^− 1^	4 × 10^− 1^	4 × 10^− 1^	5 × 10^− 1^

### Example 2

Suppose $\lambda=-2$, while the neurons are selected as 15:

15 $$\left\{\begin{array}{c}\frac{{d}^{2}p\left(u\right)}{d{u}^{2}}=2{p}^{3}\left(u\right)+up\left(u\right)-2,\\p\left(0\right)=\frac{dp\left(0\right)}{du}=0.\end{array}\right.$$

A fitness function is presented as:

16 $${E}_{Fit}=\frac{1}{15}{\left(\frac{{d}^{2}}{d{u}^{2}}{\widehat{p}}_{i}-2{\widehat{p}}_{i}^{3}-{u}_{i}{\widehat{p}}_{i}+2\right)}^{2}+\frac{1}{2}\left(({\widehat{p}}_{0}{)}^{2}+{\left(\frac{d}{du}{\widehat{p}}_{0}\right)}^{2}\right).$$

The numerical performances using the optimization of GNN-PSO-SQP are presented for the optimization of Eq. (16). These weights characterize the fit parameters, which perform the suitable outcomes approximation. Fifteen runs in Example 2 are accomplished that presents the calculations for numerous neuronal calculations to confirm the designed scheme for defining the neurons based on optimal fitness values. The GF is applied as an error function, given as:

17 $$\widehat{p}\left(u\right)={\sum}_{i=1}^{15}{k}_{i}(2{{tan}}^{-1}{e}^{({w}_{i}u+{j}_{i})}-\frac{1}{2}\pi).$$

The efficient form of above system is given as:

18 $$\begin{aligned}\widehat{p}\left(u\right)=2.7517(2{{tan}}^{-1}{e}^{(-2.8275u+5.9060)}\\-\frac{1}{2}\pi)+0.4706(2{{tan}}^{-1}{e}^{(1.5063u+0.8777)}\\-\frac{1}{2}\pi)-1.2509(2{{tan}}^{-1}{e}^{(0.4563u+2.4381)}\\-\frac{1}{2}\pi)+0.9920(2{{tan}}^{-1}{e}^{(0.0812u-0.5314)}-\frac{1}{2}\pi)\\-0.3904(2{{tan}}^{-1}{e}^{(2.7632u-2.9747)}\\-\frac{1}{2}\pi)-0.5813(2{{tan}}^{-1}{e}^{(1.5770u-1.9717)}-\frac{1}{2}\pi)\\-1.0076(2{{tan}}^{-1}{e}^{(1.6176u-1.3906)}\\-\frac{1}{2}\pi)-0.0982(2{{tan}}^{-1}{e}^{(-0.5303u+1.6984)}-\frac{1}{2}\pi)\\-1.1783(2{{tan}}^{-1}{e}^{(0.4696u+1.5836)}\\-\frac{1}{2}\pi)+3.4144(2{{tan}}^{-1}{e}^{(2.0295u-3.0027)}-\frac{1}{2}\pi)-1.9040(2{{tan}}^{-1}{e}^{(0.0495u+1.9824)}\\-\frac{1}{2}\pi)+0.9467(2{{tan}}^{-1}{e}^{(1.6989u+1.7632)}-\frac{1}{2}\pi)\\-1.0553(2{{tan}}^{-1}{e}^{(0.4236u+2.0104)}-\frac{1}{2}\pi)-1.3356(2{{tan}}^{-1}{e}^{(0.1972u+1.6735)}-\frac{1}{2}\pi).\end{aligned}$$

**Fig. 4:**
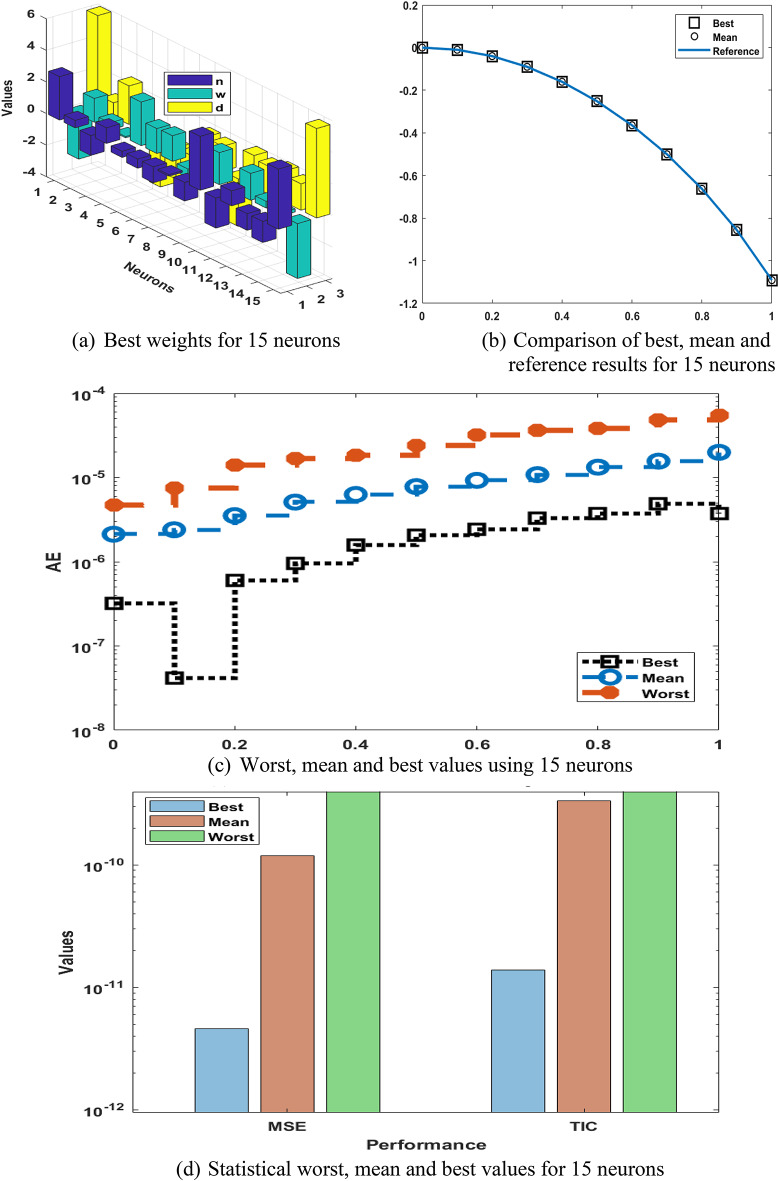
Best weights, outputs comparisons, AE, and numerical outcomes for differential PM-II-NO using GNN-PSO-SQP based 15 neurons.

**Fig. 5:**
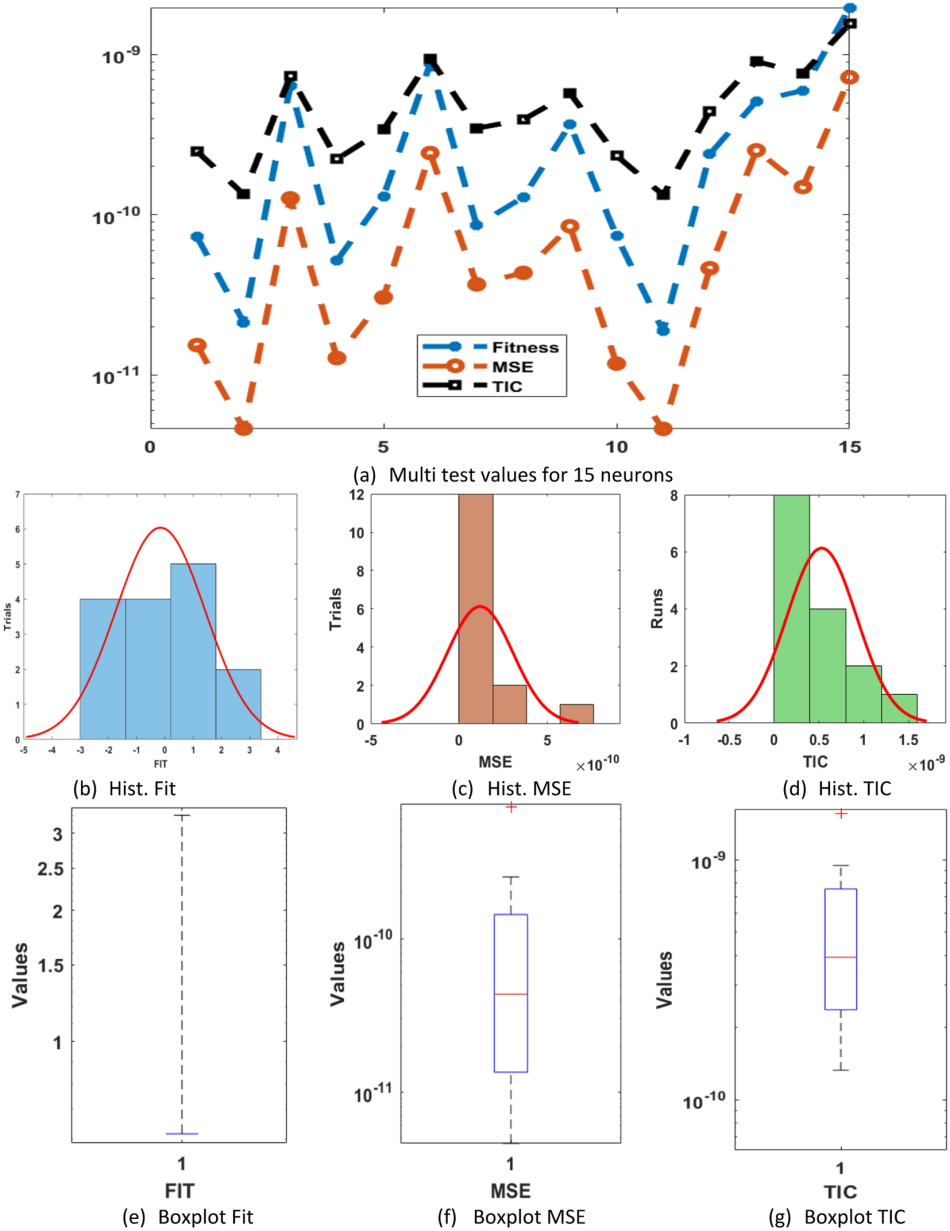
Multi test values, Hist. and boxplot for the differential PM-II-NO using GNN-PSO-SQP based 15 neurons.

The optimal values of the weights, assessment of outputs, AE and numerical outputs for differential PM-II-NO using the GNN-PSO-SQP are provided in Fig. 4. The best values of the weights have been presented in Fig. 4(a) using 15 trials. The matching of mean and best solutions is provided based on the comparison of results in Fig. 4(b). It is perceived that best and mean results in 15 trials have coincided with reference outcomes, which presents the aptitude of designed scheme to indicate the results of differential PM-II-NO using the GNN-PSO-SQP. The AE performances worst, mean and best solutions are signified in Fig. 4(c). The worst, mean, and best outcome values are calculated as 10^− 04 ^- 10^− 05^, 10^− 05^ - 10^− 06^, and 10^− 06^ - 10^− 08^. These values of AE using 15 neurons are improved in comparison to five neurons. Even the mean values for the five numbers of neurons are not similar to the reference values. However, the mean, worst, and best results are accurately performed for fifteen numbers of neurons. The statistical worst, mean, and best performances based on 15 neurons are obtainable in Fig. 4(d). The MSE and TIC values also accomplished insignificant improved in comparison with 15 neurons. The tests for worst, mean and best MSE values are performed as 10^− 04^ to 10^− 05^, 10^–10^ to 10^–11^, and 10^–11^ to 10^–12^, while the worst, mean and best TIC performances are reported as 10^− 05^ to 10^− 06^, 10^− 09^ to 10^–10^, and 10^–10^ to 10^–11^. A similar representation has been stated in Fig. 5 that presents more accurate results compared with 5 neurons. Based on these outputs, it is claimed that the trustworthiness and steadiness are somewhat better for 15 neurons as compared to five neurons. Table 3 presents the values-based Min, MD, mean, SIR, and STD values, which are found to be better as compared to 5 neurons.

**Table 3:** Statistical performances using GNN-PSO-SQP for the differential PM-II-NO.

Mode	$\widehat{p}\left(u\right)$										
	0	0.1	0.2	0.3	0.4	0.5	0.6	0.7	0.8	0.9	1
Min	8 × 10^− 8^	4 × 10^− 8^	6 × 10^− 7^	1 × 10^− 6^	1 × 10^− 7^	2 × 10^− 6^	2 × 10^− 6^	2 × 10^− 6^	5 × 10^− 7^	3 × 10^− 6^	4 × 10^− 6^
MD	6 × 10^− 7^	1 × 10^− 6^	3 × 10^− 6^	4 × 10^− 6^	4 × 10^− 6^	6 × 10^− 6^	7 × 10^− 6^	8 × 10^− 6^	1 × 10^− 5^	1 × 10^− 5^	1 × 10^− 5^
Mean	2 × 10^− 6^	2 × 10^− 6^	4 × 10^− 6^	5 × 10^− 6^	6 × 10^− 6^	8 × 10^− 6^	9 × 10^− 6^	1 × 10^− 5^	1 × 10^− 5^	2 × 10^− 5^	2 × 10^− 5^
SIR	1 × 10^− 6^	8 × 10^− 7^	1 × 10^− 6^	2 × 10^− 6^	3 × 10^− 6^	3 × 10^− 6^	4 × 10^− 6^	4 × 10^− 6^	8 × 10^− 6^	7 × 10^− 6^	1 × 10^− 5^
STD	3 × 10^− 6^	3 × 10^− 6^	4 × 10^− 6^	5 × 10^− 6^	6 × 10^− 6^	6 × 10^− 6^	8 × 10^− 6^	9 × 10^− 6^	1 × 10^− 5^	1 × 10^− 5^	1 × 10^− 5^

### Example 3

Suppose $\lambda=-2$, while the neurons are selected as 45:

19 $$\left\{\begin{array}{c}\frac{{d}^{2}p\left(u\right)}{d{u}^{2}}=2{p}^{3}\left(u\right)+up\left(u\right)-2,\\p\left(0\right)=\frac{dp\left(0\right)}{du}=0.\end{array}\right.$$

A fitness function is presented as:

20 $${E}_{Fit}=\frac{1}{45}{\left(\frac{{d}^{2}}{d{u}^{2}}{\widehat{p}}_{i}-2{\widehat{p}}_{i}^{3}-{u}_{i}{\widehat{p}}_{i}+2)\right)}^{2}+\frac{1}{2}\left(({\widehat{p}}_{0}{)}^{2}+{\left(\frac{d}{du}{\widehat{p}}_{0}\right)}^{2}\right).$$

The numerical results using the optimization of GNN-PSO-SQP are signified for the optimization Eq. (20). These performances of the weights show fit parameters, which perform a suitable approximation of the outcomes. A total of 45 runs in Example 3 is accomplished that presents the calculations for numerous neuronal calculations to confirm the scheme for defining the neurons based on optimal-fitting. The GF is applied as an error function, given as:

21 $$\widehat{p}\left(u\right)={\sum}_{i=1}^{45}{k}_{i}(2{{tan}}^{-1}{e}^{({w}_{i}u+{j}_{i})}-\frac{1}{2}\pi).$$

The rationalized structure of the above system becomes as:

22 $$\begin{aligned}\widehat{p}\left(u\right)=1.9716(2{{tan}}^{-1}{e}^{(-0.1011u-0.7915)}-\frac{1}{2}\pi)-1.2648(2{{tan}}^{-1}{e}^{(0.3429u+1.6947)}\\-\frac{1}{2}\pi)+\dots+0.0012(2{{tan}}^{-1}{e}^{(-0.8833u-0.4328)}\\-\frac{1}{2}\pi)+1.6262(2{{tan}}^{-1}{e}^{(0.3614u-0.8124)}-\frac{1}{2}\pi)+1.4036(2{{tan}}^{-1}{e}^{(0.2177u-1.0811)}-\frac{1}{2}\pi).\end{aligned}$$

**Fig. 6:**
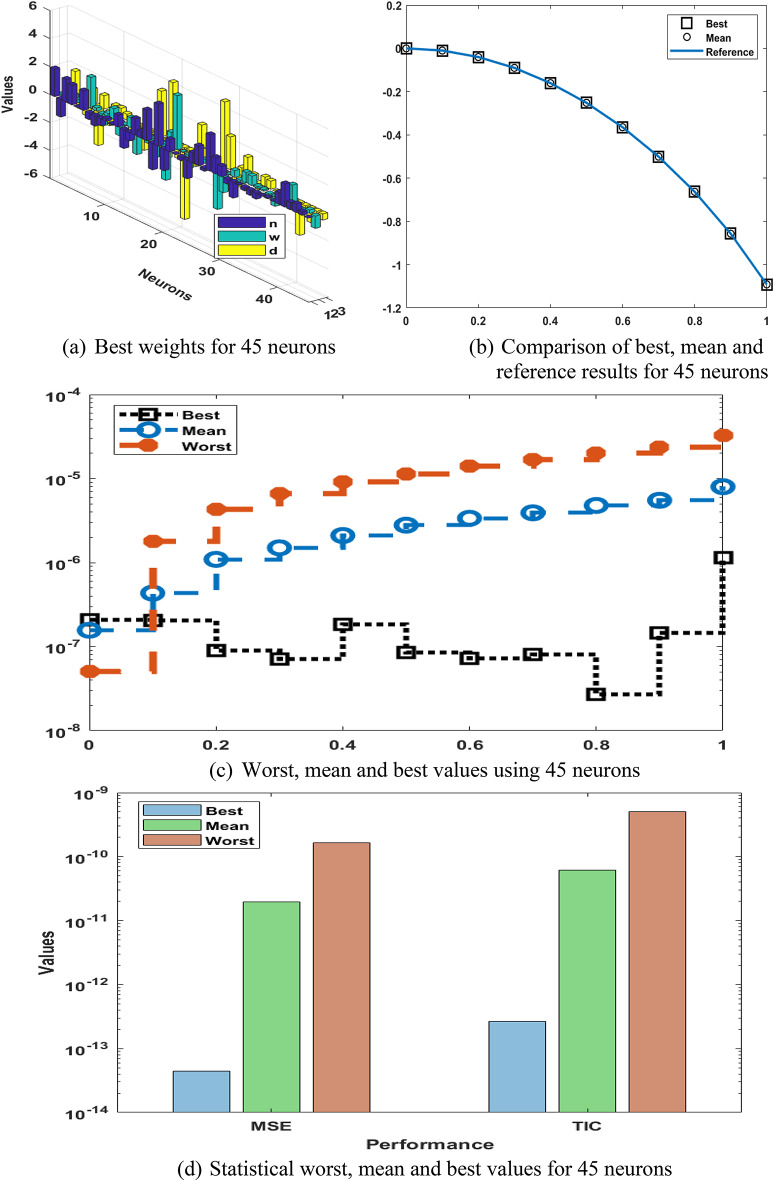
Weights, output comparisons, AE, and performances for differential PM-II-NO using GNN-PSO-SQP based 45 neurons.

**Fig. 7:**
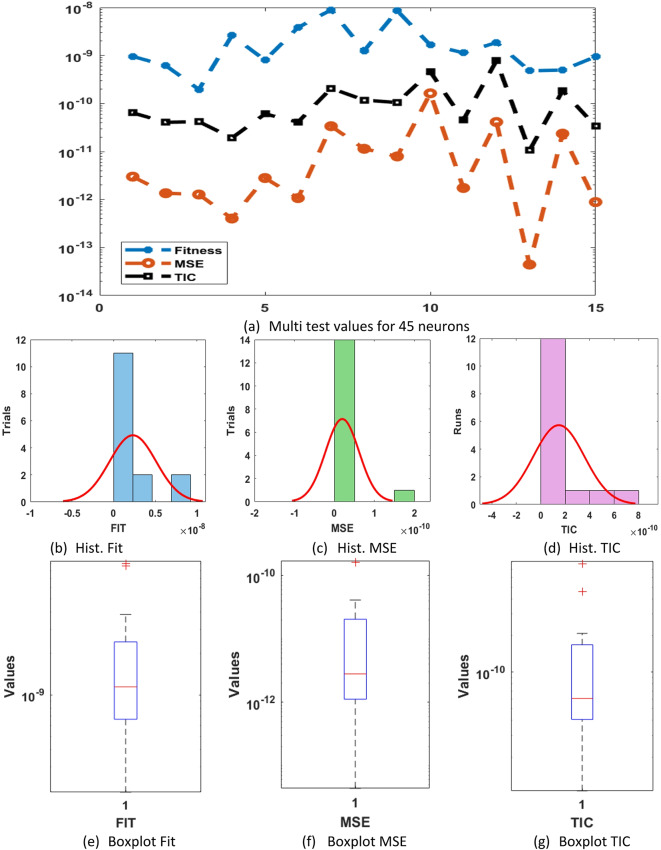
Multi test values, Hist. and boxplot for the differential PM-II-NO using GNN-PSO-SQP based 45 neurons.

The weights, output comparisons, AE and numerical outcomes for differential PM-II-NO using the GNN-PSO-SQP are reported in Fig. 6 and statistical data based 45 trials are shown in Fig. 7. Based on these outcomes, it is measured the meticulousness and steadiness is gotten improved for 45 neurons in comparison with 5 and 15. It is shown that by enhancing neurons, the scheme’s performance is obtained better to solve the differential PM-II-NO using the GNN-PSO-SQP. Table 4 shows the outcomes based on 45 neurons that present more accurate values in comparison with 5 and 15 neurons for solving the differential PM-II-NO using the GNN-PSO-SQP.

**Table 4:** Statistical performances using GNN-PSO-SQP for the differential PM-II-NO.

Mode	$\widehat{p}\left(u\right)$										
	0	0.1	0.2	0.3	0.4	0.5	0.6	0.7	0.8	0.9	1
Min	1 × 10^− 08^	4 × 10^− 08^	9 × 10^− 08^	7 × 10^− 08^	2 × 10^− 07^	9 × 10^− 08^	7 × 10^− 08^	8 × 10^− 08^	3 × 10^− 08^	1 × 10^− 07^	5 × 10^− 07^
MD	5 × 10^− 08^	2 × 10^− 07^	7 × 10^− 07^	8 × 10^− 07^	1 × 10^− 06^	2 × 10^− 06^	2 × 10^− 06^	2 × 10^− 06^	3 × 10^− 06^	3 × 10^− 06^	5 × 10^− 06^
Mean	2 × 10^− 07^	4 × 10^− 07^	1 × 10^− 06^	1 × 10^− 06^	2 × 10^− 06^	3 × 10^− 06^	3 × 10^− 06^	4 × 10^− 06^	5 × 10^− 06^	6 × 10^− 06^	8 × 10^− 06^
SIR	1 × 10^− 07^	2 × 10^− 07^	5 × 10^− 07^	8 × 10^− 07^	1 × 10^− 06^	1 × 10^− 06^	2 × 10^− 06^	2 × 10^− 06^	3 × 10^− 06^	3 × 10^− 06^	6 × 10^− 06^
STD	2 × 10^− 07^	4 × 10^− 07^	1 × 10^− 06^	2 × 10^− 06^	2 × 10^− 06^	3 × 10^− 06^	4 × 10^− 06^	4 × 10^− 06^	5 × 10^− 06^	6 × 10^− 06^	9 × 10^− 06^

The performance based on the cost of complexity cost performances for solving the differential PM-II-NO using GNN-PSO-SQP for Examples 1, 2, and 3 are presented in Table 5. The number of iterations performed time, and the function counts are provided in this Table. It is observed that the iteration, time, and count of the function took less time, for example 1, then it enhanced for example 2, and more time is calculated for example 3. It shows that the complexity cost is calculated as high for 45 neurons as compared to 15 and 5 neurons.

**Table 5:** Different performances for each Example of the PM-II-NO using GNN-PSO-SQP.

Example	Iterations		Performed Time		Function Computations	
	Mean	STD	Mean	STD	Mean	STD
1	2.3164294	4.3216964	0.7036026	0.1000002	2.946515	3.8930663
2	43.1147524	14.1792967	1505	3.5097787	122423.7333333	35203.4708627
3	594.7357001	149.0158344	1436.0000000	267.2358509	392044.9333333	73245.9005869

## Conclusion

In present research, the investigations of the differential PM-II-NO have been numerically presented by designing a novel GNN. The mathematical PM-II-NO form is a second-order nonlinear differential system, which is handled by the designed computing structure. The construction of the error function has been performed through the differential PM-II-NO and its ICs. The optimization of the procedure is perceived through the hybridization of local and global search terminologies. The comparison of the proposed and literature solutions is also provided to authenticate the exactness of the designed solver. The combination of global search PSO and local search SQP has been applied to get the efficient results of the differential PM-II-NO. The procedure’s exactness has been performed via matching of results and insignificant AE, which are calculated as 10^− 05^ to 10^− 07^. Statistical tests using multiple independent executions have also been achieved to authenticate the consistency of the designed GNN-PSO-SQP for the differential PM-II-NO. The neuron analysis based on 5, 15, and 45 numbers of neurons has been performed in order to examine the analysis of neurons. The higher numbers of neurons performed better as compared to smaller neuron values; however, the complexity cost of larger numbers of neurons is calculated more as compared of smaller numbers of neurons.

### Future research direction

The proposed GNN-PSO-SQP can be applied to solve thermal and mass transfer in pumping flow of Ellis fluid model ^39^, Lorenz differential equations ^40^, mixed convection flow in an inclined tube with ciliary motion of Jeffrey six constant fluid ^41^, and multiphase peristaltic flow model ^42^.

## Data Availability

The datasets used and/or analyzed during the current study are available from the corresponding author on reasonable request.

## References

[1] Kruskal, M. D. Analytic and asymptotic methods for nonlinear singularity analysis: A review and extensions of tests for the Painlevé property. In *Integrability of Nonlinear Systems* 175–208 (2004).

[2] Palese, M. et al. Particle-like, dyx-coaxial and trix-coaxial Lie algebra structures for a multi-dimensional continuous Toda type system. *Nucl. Phys. B* **960**, 115187 (2020).

[3] Joshi, N. et al. Geometric description of a discrete power function associated with the sixth Painlevé equation. *Proc. R. Soc. A Math. Phys. Eng. Sci.* **473**(2207), 20170312 (2017). 10.1098/rspa.2017.0312 PMC5719623 29225492

[4] Blower, G. On linear systems and τ functions associated with Lamé’s equation and Painlevé’s equation VI. *J. Math. Anal. Appl.* **376** (1), 294–316 (2011).

[5] Bermudez, D. et al. January. Supersymmetric quantum mechanics and Painlevé equations. In AIP Conference Proceedings (Vol. 1575, 1, 50–88). American Institute of Physics. (2014).

[6] Wazwaz, A. M. et al. On the Painlevé integrability and nonlinear structures to a (3 + 1)-dimensional Boussinesq-type equation in fluid mediums: Lumps and multiple soliton/shock solutions. *Phys. Fluids*10.1063/5.0194071 (2024).

[7] Mohan, B. et al. Painlevé analysis, restricted bright-dark N-solitons, and N-rogue waves of a (4 + 1)-dimensional variable-coefficient generalized KP equation in nonlinear sciences. *Nonlinear Dyn.* **113** (10), 11893–11906 (2025).

[8] Akter, F. et al. A comprehensive review of mathematical modeling for drying processes of fruits and vegetables. *Int. J. Food Sci.* **2022**(1), 6195257 (2022). 35910694 10.1155/2022/6195257 PMC9334071

[9] Sahai, N. et al. Mathematical modeling and simulations for developing nanoparticle-based cancer drug delivery systems: A review. *Curr. Pathobiol. Rep.* **9**, 1–8 (2021).

[10] Veeresha et al. Regarding on the fractional mathematical model of Tumour invasion and metastasis. *Comput. Model. Eng. Sci.* **127** (3), 1013–1036 (2021).

[11] Ullah, G. W. et al. A multi-objective mathematical model of a water management problem with environmental impacts: An application in an irrigation project. *PLoS One* **16**(8), e0255441 (2021). 34343172 10.1371/journal.pone.0255441 PMC8330924

[12] Aba et al. A fractional order mathematical model for COVID-19 dynamics with quarantine, isolation, and environmental viral load. Advances in Difference Equations, 2021,1–19. (2021). 10.1186/s13662-021-03265-4 PMC7877321 33613668

[13] Lun, Y. et al. December. Experimental study and suggested mathematical model for chloride-induced reinforcement corrosion rate. In Structures (Vol. 34, 2014–2029). Elsevier. (2021).

[14] Sana, S. S. A structural mathematical model on two echelon supply chain system. *Ann. Oper. Res.* **315**(2), 1997–2025 (2022).

[15] Sinan, M. et al. Fractional mathematical modeling of malaria disease with treatment & insecticides. *Results Phys.* **34**, 105220 (2022).

[16] Ahmad, I. et al. Neuro-evolutionary computing paradigm for Painlevé equation-II in nonlinear optics. *Eur. Phys. J. Plus* **133**, 1–15 (2018).

[17] Faridi, W. A. et al. The Lie point symmetry criteria and formation of exact analytical solutions for Kairat-II equation: Paul-Painlevé approach. *Chaos. Solitons. Fractals.* **182**, 114745 (2024).

[18] Fornberg, B. et al. A computational exploration of the second Painlevé equation. *Found Comput Math.* **14**, 985–1016 (2014).

[19] Sabir, Z. et al. Solution of fractional order mathematical lungs cancer operation model: A radial basis neural network. *Netw. Model. Anal. Health Inf. Bioinf.* **14**(1), 1–13 (2025).

[20] Sabir, Z. et al. A machine learning radial basis Bayesian regularization neural network procedure for the cholera disease model. *Netw. Model. Anal. Health Inf. Bioinf.* **14** (1), 1–16 (2025).

[21] Sabir, Z. et al. A novel combination of sigmoid and radial basis neural networks for the monkeypox transmission system. *Eng. Appl. Artif. Intell.* **158**, 111512 (2025).

[22] Sabir, Z. et al. A radial basis scale conjugate gradient neural network process for the Zika model with human movement and reservoirs. *Chaos Solitons Fractals* **199**, 116711 (2025).

[23] Sabir, Z. et al. Numerical treatment of fractional order Buruli ulcer and cholera model by using neural network approach. *Knowl. Based Syst.* **320**, 113713 (2025).

[24] Saqib, S. U., Shih, Y. T., Anjum, M. W. & Shoaib, M. *Advanced Heuristic Computing with Gudermannian Neural Networks for Mathematical Modeling of Divorced Dynamics in Social Networks* (Mathematics and Computers in Simulation, 2025).

[25] Saqib, S. U., Shih, Y. T. & Anjum, M. W. A metaheuristic approach integrating neuro-evolutionary genetic and Sqp with Gudermanian neural networks for precision cattle skin disease. Available at SSRN 5209410.

[26] Lee, I. H., Saqib, S., Sheng, Q. & Shih, Y. T. An epidemiological model of the mumps virus via Mittag-Leffler kernel: Stability analysis and artificial neural network solutions. *AIMS Math.* **10**(10), 24923–24957 (2025).

[27] Saqib, S. U., Shih, Y. T., Jahanzaib, M., Wahab, A. & Shih-Hau, F. Application of tri-layered RNN scheme for Maxwell model subject to MHD. *AIMS Math.* **11**(1), 881 (2026).

[28] Faisal, S., Sabir, Z., Khan, S. U., Aamir, M. & Cyran, K. A. A novel heuristic Morlet wavelet neural network design for the Painlevé equation-II arising in nonlinear optics. *Sci. Rep.* **15**(1), 24713 (2025). 40634666 10.1038/s41598-025-10096-w PMC12241396

[29] Tiwari, S. & Kumar, A. Advances and bibliographic analysis of particle swarm optimization applications in electrical power system: Concepts and variants. *Evol. Intel.* **16**(1), 23–47 (2023).

[30] Zhang, Y. Elite archives-driven particle swarm optimization for large scale numerical optimization and its engineering applications. *Swarm Evol. Comput.* **76**, 101212 (2023).

[31] Pozna, C., Precup, R. E., Horváth, E. & Petriu, E. M. Hybrid particle filter–particle swarm optimization algorithm and application to fuzzy controlled servo systems. *IEEE Trans. Fuzzy Syst.* **30** (10), 4286–4297 (2022).

[32] Almutairi, A. F., Al-Gharabally, M. & Salman, A. A. Particle swarm optimization application for multiple attribute decision making in vertical handover in heterogenous wireless networks. *J. Eng. Res.*10.36909/jer.v9i1.10331 (2021).

[33] Kuo, R. J., Luthfiansyah, M. F., Masruroh, N. A. & Zulvia, F. E. Application of improved multi-objective particle swarm optimization algorithm to solve disruption for the two-stage vehicle routing problem with time windows. *Expert Syst. Appl.* **225**, 120009 (2023).

[34] Fliege, J. & Vaz, A. I. F. A method for constrained multiobjective optimization based on SQP techniques. *SIAM J. Optim.* **26** (4), 2091–2119 (2016).

[35] Gill, E., Murray, W. & Saunders, M. A. SNOPT: An SQP algorithm for large-scale constrained optimization. *SIAM Rev.* **47**(1), 99–131 (2005).

[36] Hermana, M., Ngui, J. Q., Weng Sum, C. & Prasad Ghosh, D. Feasibility study of SQp and SQs attributes application for facies classification. *Geosciences* **8**(1), 10 (2018).

[37] Jiang, H. & Ralph, D. Smooth SQP methods for mathematical programs with nonlinear complementarity constraints. *SIAM J. Optim.* **10** (3), 779–808 (2000).

[38] Thanedar, B., Arora, J. S., Tseng, C. H., Lim, O. K. & Park, G. J. Performance of some SQP algorithms on structural design problems. *Int. J. Numer. Methods Eng.* **23** (12), 2187–2203 (1986).

[39] Aslam, M. N., Riaz, A., Shehzadi, M., Akram, S. & Bhatti, M. M. Computational analysis for thermal and mass transfer in pumping flow of Ellis fluid along with solid particles: Morlet wavelet neural networks approach. *Eng. Appl. Artif. Intell.* **155**, 111096 (2025).

[40] Aslam, M. N. et al. Neuro-computing solution for Lorenz differential equations through artificial neural networks integrated with PSO-NNA hybrid meta-heuristic algorithms: A comparative study. *Sci. Rep.* **14**(1), 7518 (2024). 38553496 10.1038/s41598-024-56995-2 PMC11344159

[41] Aslam, M. N. et al. An ANN-PSO approach for mixed convection flow in an inclined tube with ciliary motion of Jeffrey six constant fluid. *Case Stud. Therm. Eng.* **52**, 103740 (2023).

[42] Alqudah, M. et al. Thermal and mass exchange in a multiphase peristaltic flow with electric-debye-layer effects and chemical reactions using machine learning. *Case Stud. Therm. Eng.* **56**, 104234 (2024).

